# JAK/Stat5-mediated subtype-specific lymphocyte antigen 6 complex, locus G6D (LY6G6D) expression drives mismatch repair proficient colorectal cancer

**DOI:** 10.1186/s13046-018-1019-5

**Published:** 2019-01-22

**Authors:** Guido Giordano, Pietro Parcesepe, Mario Rosario D’Andrea, Luigi Coppola, Tania Di Raimo, Andrea Remo, Erminia Manfrin, Claudia Fiorini, Aldo Scarpa, Carla Azzurra Amoreo, Fabiana Conciatori, Michele Milella, Francesca Pia Caruso, Luigi Cerulo, Almudena Porras, Massimo Pancione

**Affiliations:** 1 Oncology Unit, Casa Sollievo della Sofferenza-IRCCS, San Giovanni Rotondo, Italy; 2 Medical Oncology and Anatomic Pathology Unit, San Filippo Neri Hospital, Rome, Italy; 3 Department of Diagnostics and Public Health – Section of Pathology, University and Hospital Trust of Verona, Verona, Italy; 4 Pathology Unit, “Mater Salutis” Hospital AULSS9, Legnago (Verona), Italy; 5 Pathology, IRCCS Regina Elena National Cancer Institute, Rome Italy, Via Elio Chianesi 53, 00144 Rome, Italy; 6 Medical Oncology, IRCCS Regina Elena National Cancer Institute, Rome Italy, Via Elio Chianesi 53, 00144 Rome, Italy; 7 Department of Sciences and Technologies, University of Sannio, Via Port’Arsa, 1182100 Benevento, Italy; 8 Bioinformatics Laboratory, BIOGEM scrl, Ariano Irpino, Avellino, Italy; 9 Department of Biochemistry and Molecular Biology, Faculty of Pharmacy, Complutense University Madrid, Madrid, Spain; 10 Health Research Institute of the Hospital Clínico San Carlos (IdISSC), Madrid, Spain

**Keywords:** LY6G6D, Colorectal cancer, Microsatellite-stable, Immune resistance

## Abstract

**Background:**

Human microsatellite-stable (MSS) colorectal cancers (CRCs) are immunologically “cold” tumour subtypes characterized by reduced immune cytotoxicity. The molecular linkages between immune-resistance and human MSS CRC is not clear.

**Methods:**

We used transcriptome profiling, in silico analysis, immunohistochemistry, western blot, RT-qPCR and immunofluorescence staining to characterize novel CRC immune biomarkers. The effects of selective antagonists were tested by in vitro assays of long term viability and analysis of kinase active forms using anti-phospho antibodies.

**Results:**

We identified the lymphocyte antigen 6 complex, locus G6D (LY6G6D) as significantly overexpressed (around 15-fold) in CRC when compared with its relatively low expression in other human solid tumours. LY6G6D up-regulation was predominant in MSS CRCs characterized by an enrichment of immune suppressive regulatory T-cells and a limited repertoire of PD-1/PD-L1 immune checkpoint receptors. Coexpression of LY6G6D and CD15 increases the risk of metastatic relapse in response to therapy. Both JAK-STAT5 and RAS-MEK-ERK cascades act in concert as key regulators of LY6G6D and Fucosyltransferase 4 (*FUT4),* which direct *CD15*-mediated immune-resistance. Momelotinib, an inhibitor of JAK1/JAK2, consistently abrogated the STAT5/LY6G6D axis in vitro*,* sensitizing MSS cancer cells with an intact JAK-STAT signaling, to efficiently respond to trametinib, a MEK inhibitor used in clinical setting. Notably, colon cancer cells can evade JAK2/JAK1-targeted therapy by a reversible shift of the RAS-MEK-ERK pathway activity, which explains the treatment failure of JAK1/2 inhibitors in refractory CRC.

**Conclusions:**

Combined targeting of STAT5 and MAPK pathways has superior therapeutic effects on immune resistance. In addition, the new identified LY6G6D antigen is a promising molecular target for human MSS CRC.

**Electronic supplementary material:**

The online version of this article (10.1186/s13046-018-1019-5) contains supplementary material, which is available to authorized users.

## Background

Colorectal cancer (CRC) development involves complex interactions between malignant cells and immune system, not comprehensively defined [1]. Targeted therapies have improved patient outcomes. However, multiple drug resistance mechanisms often converge to reactivate the original pathway targeted by these drugs or alternatively compensatory kinase cascades [2]. Mutations in Mitogen-activated protein kinase (MAPK) cascade, phosphoinositide 3-kinase CA (*PIK3CA)* or aberrant activation of tyrosine kinase receptors (HER3 or MET) function as prominent factors of resistance [3, 4].

In CRC patients, only a modest clinical effect of MAPK inhibitors has been reported. In this context, studies have shown that CD15, also called Lewis^x^ antigen, synthetized by Fucosyltransferase 4 (*FUT4),* is induced by the RAF-MEK-ERK signaling pathway, and colon cancers that are FUT4+/CD15+ seem to exhibit significant alteration of the systemic immune surveillance and resistance to the anti-EGFR agents (cetuximab) [5]. This mechanism blocks cytotoxic T lymphocyte activities against tumour cells, making malignant cells progressively more aggressive and difficult to treat [6, 7]. It is well known that microsatellite instability (MSI) and mismatch repair (MMR) defects can lead to DNA hypermutation and the production of immunogenic neo-peptides, recognized by antigen-specific tumour infiltrating lymphocytes, which is counterbalanced by the upregulation of multiple immune checkpoint molecules [8–10]. These tumours are characterized by a predominant type of T helper cells (Th) with Th1 phenotype (Th1), which potentiate the lytic function of cytotoxic effector T cells present in the tumor microenvironment, activating *IFNγ, IL-15* and JAK (Janus kinase)/STAT (signal transducer and activator of transcription) pathways [11, 12]. Tumours defective in MMR machinery represent only 5% of all metastatic colorectal cancers and they are more easily recognized by the immune system [13].

The Food and Drug Administration (FDA) has recently approved the checkpoint inhibitor anti-Programmed cell death protein 1 (PD1) for the treatment of metastatic MMR defective CRC, when the disease has progressed after chemotherapy [13]. Unfortunately, mutations in (JAK1/JAK2) or class I MHC molecules (TAP2, B2M) and other still unknown signaling molecules can promote an inadequate immune response against tumours [14, 15]. In addition, the identity of tumour-intrinsic immune antigens that interfere with cancer immunogenicity and antitumour T cell responses in MMR proficient tumours are poorly understood. The lymphocyte antigen 6 complex, locus G6D (LY6G6D) belongs to a cluster of leukocyte antigens located in the major histocompatibility complex (MHC) class III region on chromosome 6 [16]. LY6G is a small protein attached to the cell surface by a glycosylphosphatidylinositol (GPI) anchor, employed as a marker to identify granulocytes and myeloid-derived suppressor cell subpopulations in mouse [16]. LY6G family members might be useful as cancer vaccines and drug conjugated antibodies, but their relevance in human diseases remains enigmatic [16–18].

We here used in silico approaches, expression profiling and in vitro functional assays to characterize novel cancer-specific immune antigens in poorly immunogenic colon cancer subtypes. Our data identify LY6G6D antigen as a potential molecular target for human microsatellite stable tumours and provide evidences supporting that a combined targeting of MAPK and STAT5 signaling can improve the therapeutic response in this subtype.

## Methods

Materials and Methods and any associated references are described more in detail within the Additional file 1.

### Gene expression data analysis among different tumor subtypes

The Gene Expression Profile from 604 cancer cell lines representative of 14 different tumor sites from the Cancer Cell Lines Encyclopedia series were analyzed. We selected a collection of ~ 6000 known human genes with immunomodulatory functions from InnateDB, Innate Immunity Genes curated database (http://allergen.innatedb.com/). ANOVA analysis was adopted to test for differential expression among different tumor subtypes while eta squared was used to determine those with a greater effect size. A series of 55 colorectal cancer cell lines from the Cancer Cell Lines Encyclopedia was selected to visualize DNA copy number and mutational load. A total of 17 cancer types were retrieved from The Cancer Genome Atlas (TCGA) dataset to analyze gene expression levels.

### Immune cell type enrichment analysis

To analyze the expression changes related to specific immune cell subpopulations, we applied a deconvolution approach based on Gene Set Enrichment Analysis (GSEA). Unsupervised hierarchical clustering was then applied on the Euclidean distance and Ward linkage method on the matrix of the enrichment scores.

### Patient samples and tissue microarrays analysis

Two independent datasets of patients with sporadic CRC were retrospectively recruited and collected (Additional file 2: Table S1 and Table S2). Additional samples included sections of fresh tissue specimens from tumor and matched normal adjacent mucosa frozen in liquid nitrogen. The recruitment of the patients was performed in accordance with the ethical guidelines, protocol number: 1703/2016 of September 2016 from the San Filippo Neri Hospital, Rome, Italy. The tissue microarrays (TMAs) used for this study included tumor tissue from 516 unselected colon carcinoma and 92 corresponding normal mucosa specimens. Construction of the TMAs has been previously described [5].

### Immune localization and western blot analysis

TMA slides were stained individually with horseradish peroxidase-conjugated avidin biotin complex (ABC) as previously reported [5]. Infiltrating immune cells were counted automatically by using ImageJ-based software. Whole tissue sections were used for double immunofluorescence analysis.

### Cell lines, drugs and proliferation assay

Human colon cancer cell lines were purchased from American Type Culture Collection (ATCC, Rockville, MD, USA) or kindly donated from other laboratories and they were cultured as described [5]. All cell lines were mycoplasma free. Each drug was diluted in culture medium, just before each experiment. The data from the Genomics of Drug Sensitivity in Cancer project (Sanger panel) were retrieved for more of 30 colon cancer cell lines. A set of 481 small-molecules that collectively modulate a broad array of cell processes (https://portals.broadinstitute.org/ctrp/) was used to identify colon cancer dependencies to inhibitor molecules. Cell proliferation/survival was measured using the 3-(4,5-dimethylthiazol-2-yl)-2,5- diphenyltetrazolium bromide (MTT). The IC50 was determined by interpolation from the dose response curves. Long term viability following drug treatments was assessed by colony formation assays.

### *Analysis of Kinases active forms*, RNA interference and quantification of mRNA by RT-qPCR

The analysis of Kinases in its active form was performed by using specific antiphospho antibodies that recognized the phosphorylated active forms normalizing with to antibodies against the total protein. For RNA interference, small inhibitor specific RNAs and scrambled control were transiently transfected into cells. Reverse Transcription Real-Time Quantitative PCR (RT-qPCR) was used to quantitatively determine mRNA expression normalized to GAPDH mRNA or rRNA 18S levels.

### Statistical analysis

The statistical analyses were carried out using Prism version 4.02 (GraphPad Software, Inc), GeneSpring R/bioconductor v.12.5 and R based package.

## Results

### Upregulation of *LY6G6D* antigen in colorectal cancer

**Fig. 1:**
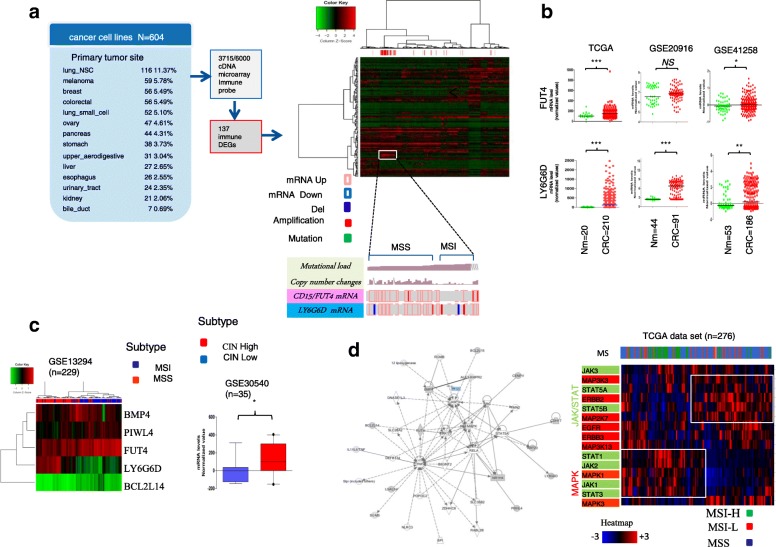
Characterization of *LY6G6D* and FUT4/*CD15* expression. **a** The work flow on the left shows cancer cell line transcriptomic samples that were retrieved from NCBI (Barretina J et al. 2102) and interrogated for differentially expressed genes of known immune-related genes from ImmPort collection. Right, unsupervised hierarchical cluster of cancer cell lines (*n* = 604) shows a gene signature enriched in colorectal cancer. Enlarged image shows two genes *LY6G6D and FUT4/CD15* within the cluster that are upregulated in Microsatellite stable (MSS) but not in microsatellite instable (MSI) colon cancer cells categorized for mutational load and copy number variations (CNVs). **b** Quantification of *CD15* and *LY6G6D* mRNA in patient-matched tumor-normal mucosa extracted from The Cancer Genome Atlas (TCGA) and Gene Expression Omnibus (GEO) data sets. Scatter plot in which each circle represents mRNA levels in each tumor sample, horizontal line is the mean value. *P ≤ 0.05; **P ≤ 0.01; ***P ≤ 0.001 by Mann–Whitney *U* test. **c** Heat map of log-transformed odds ratios of a set of immune-related genes for two different molecular phenotypes MSI vs MSS. On the left, quantification of *LY6G6D* mRNA by a box plot in CRCs classified as CIN high or low based on a weighted genome integrity index (see Methods). *P ≤ 0.05; t test Welch-corrected. **d** Enrichment map network of statistically significant gene interactions. Nodes represent gene hub and lines their connectivity. Node size is proportional to number of line with arrows. Heat map of differentially expressed genes within JAK/STAT and MAPK signaling according to MSI-H, MSI-L, MSS subtypes. Shown are groups with high relative expression (hi, red) versus the low relative expression (lo, blue) at the optimum value cutoff

We initially analyzed public transcriptome microarrays data derived from 604 human cancer cell lines [19] representative of 13 solid tumours to identify differentially expressed immune-related genes derived from Immport collection (http://www.immport.org/immport-open/public/home/home, (Fig. 1a). Gene expression profile using unsupervised hierarchical cluster analysis showed that eight genes clustered in a single branch were enriched in colon cancer as compared to other cancers (FDR < 0.01; eta squared > 0.3) (Fig. 1a and Additional file 3: Figure S1a). Notably, two genes of cluster i) a member of the lymphocyte antigen-6 (Ly6) complex, locus G6D (LY6G6D), localized on the MHC class III region (6p21) and ii) a member of the fucosyltransferase genes *(FUT4)*, encoding for the fucosylated Lewis^x^ antigen, here called CD15, as reported [5], were highly overexpressed in CRC, discriminating MSI and MSS subtypes (Fig. 1a, Additional file 3: Figure S1a).

To test the robustness of these predictions, we analyzed primary CRC samples from three independent datasets, TCGA, GSE20916 [20] and GSE41258 [21] using as a control matched normal colonic tissues. In all data sets, *LY6G6D* was highly expressed in colorectal cancer compared to normal tissues, whereas *FUT4* expression levels, tended to be significantly higher in CRC than in normal mucosa in two out of three databases (Fig. 1b). For the remaining genes of the cluster, their expression levels were lower or unchanged in CRC, compared to normal mucosa (Additional file 3: Figure S1b).

As *LY6G6D* and *FUT4* tended to be upregulated in MSS (typically poorly immunogenic), but not in MSI (typically highly immunogenic) CRC cell lines, we then analyzed other primary tumours by using GSE13294 [22] and GSE30540 [23] datasets, where MS status and chromosomal instability were available. Notably, we confirmed that *LY6G6D* expression levels were significantly higher in MSS than in MSI subset and tend to be significantly higher in chromosomal instability high (CIN-high) than in CIN-low tumours (Fig. 1c). In addition, analysis of additional datasets (*n* = 569) revealed that primary CRC surgical specimens can be discriminated in low or high-*LY6G6D*, but not on the basis of *FUT4* expression levels (Additional file 3: Figure S1c).

### *Ly6G6D* and FUT4 characterize distinct immunophenotypes in colorectal cancer

To unveil signaling pathways regulating *LY6G6D* and *FUT4*, we carried out a network analysis to look for potential interactions and regulators. We found that protein kinases, mainly MAPKs (ERKs and p38 MAPKs) and STAT5 regulated *FUT4* and *LY6G6D* expression, respectively (Fig. 1d). To establish the relevance of these findings, we determined the expression of genes from MAPKs and JAK/STAT cascades in distinct CRC subtypes (TCGA, *n* = 276) stratified as MMR status. While components of MAPK cascades were broadly expressed, the majority of JAK/STAT genes presented higher expression in MSI than in MSS tumours according with literature [13]. Notably, among JAK/STAT genes, only *STAT5* showed higher expression in MSS than in MSI and clustered with MAPK genes (Fig. 1d). Indeed, a review of exome sequencing data (*N* = 2078 CRCs from cbioportal) showed a high prevalence of JAK1/2 mutations, but not of STAT5, in hypermutated tumours [10, 14] (Additional file 3: Figure S2a).

**Fig. 2:**
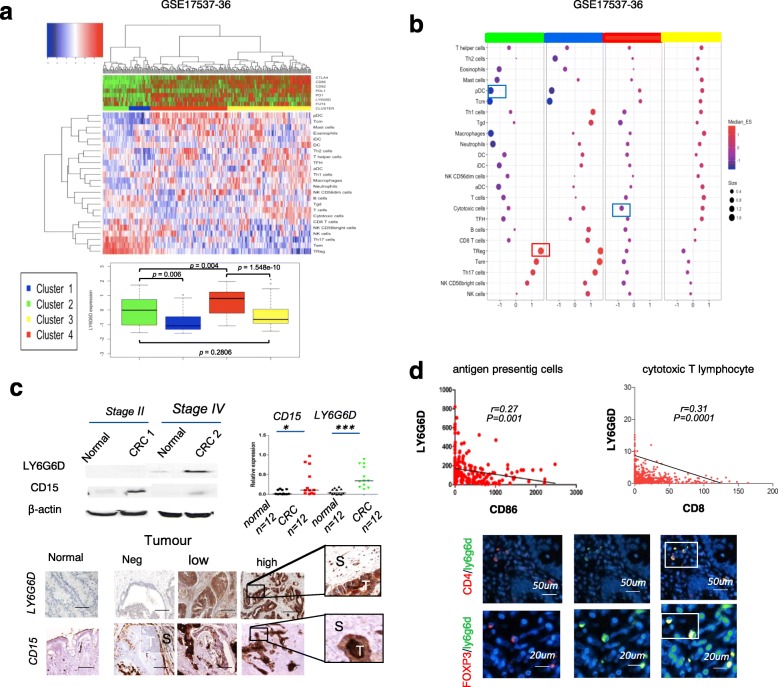
Intra-tumoral immunophenotypes marked by LY6G6D and FUT4/CD15. **a** On the top, unsupervised hierarchical cluster of 232 CRC samples (dataset: GSE17536–37) using cell-specific immune-signatures categorized patients into four groups, with distinct cell immune associated gene expression. Data are obtained using the Euclidean distance and Ward linkage method on the matrix of the enrichment scores calculated through ssGSEA. Top tracks represents the expression profile of known immune inhibitory molecules, together with LY6G6D and CD15/FUT4 genes. On the bottom, boxplots of LY6G6D gene expression in each cluster. **b** Dot plot representing the mean enrichment scores of each immune cell type in any cluster. Color scale represents the positive (red) and negative (blue) enrichment score; dot size indicates the strength of the association. **c** representative western blot images and quantification of LY6G6D and CD15 expression from CRC samples and matched normal mucosa (*n* = 12) relative to β-actin used as loading. Data are mean ± standard error of the mean (s.e.m); (*n* = 3 biological replicates, P* < 0.05, ***P < 0.001, two-tailed Student’s t-test. Low, LY6G6D and CD15 IHC in normal mucosae and tumor specimens; Scale bar, 100 μm. Enlarged is the staining in both malignant cells (T) and stromal (S) immune cells. **d** Correlation between LY6G6D+ cells, CD8 T-lymphocytes and CD86 staining in CRC samples (five replicates counts, cells mm^− 2^). Double immunofluorescence from paraffin embedded sections co-stained with antibodies against CD4 (red) and FOXP3 (red) or LY6G6D (green). Scale bar, 50 μm and 20 μm, respectively

As ERKs and JAK/STAT5 cascades have been implicated in immune evasion, we analyzed tumor-infiltrating immune cell subpopulations in relation to gene expression levels of *LY6G6D, FUT4* and others key immune modulatory molecules. Immune cell deconvolution revealed that CRCs clustered based on enrichment of distinct immune cell types (Fig. 2a and b and Additional file 3: Figure S2b).

In particular, we found that a CRC subtype characterized by high Tregs and low dendritic cells (DC) showed high expression levels of *LY6G6D* in concurrence with decreased expression levels of several immune checkpoint molecules such as CTLA-4 and PD1 [2, 3] (Fig. 2a and b Cluster 2, green). Similar observations were obtained using an independent dataset of validation. Also in this case, high levels of *LY6G6D* expression were correlated with high infiltrates of immune suppressive cell types, such as the regulatory T cells (Tregs) and T helper 2 (Th2) cells, which play a key role as mediators of antitumor immunity (Additional file 3: Figure S2c, Cluster 4, green). On the contrary, *FUT4* tended to be upregulated in malignant cells (Fig. 2c) displaying no significant correlation with specific immune cells type, consistent with literature [5]. These observations suggest that LY6G6D and FUT4 may be responsible for cancer progression acting as tumour-intrinsic immune suppressive factors.

### Coexpression of *LY6G6D* and CD15 promotes cancer progression in concert

To further investigate the role of *LY6G6D* and FUT4, we focused on CD15, a cell-surface antigen encoded by FUT4, whose over-expression in metastatic CRC is associated with lack of response to EGFR and VEGF inhibitors [5]. We studied by western blot analysis our in-house snap frozen CRCs (*n* = 12) and healthy mucosa samples (*n* = 12) obtained from the same patient. We observed increased expression of *LY6G6D* and *CD15* in primary CRC as compared with healthy mucosa. Semi-quantitative protein analysis revealed that LY6G6D, but not CD15, was consistently higher in stages III and IV than in stages I and II of primary tumours (Fig. 2c).

Next, we performed immunohistochemistry (IHC) in two independent datasets (I and II) comprising 517 surgically resected tumors and 77 normal mucosa samples (Additional file 2: Tables 1 and 2). IHC results revealed that approximately 70% of tumours expressed CD15 in malignant cells (Additional file 3: Figure S3a), which correlated with a reduction in the infiltrating neutrophils and monocytes (Fig. 2c), in line with previous findings [5]. Notably, more than 80% of tumours exhibited LY6G6D staining, compared to 20% of normal tissues (Additional file 3: Figure S3a). Accordingly, double immunofluorescence on paraffin embedded tumours revealed that CD4+ and FOXP3+ T cells (Treg) were positive for LY6G6D staining, whereas CD8+ T lymphocytes did not (Fig. 2d and Additional file 3: Figure S3b)*.*

**Fig. 3:**
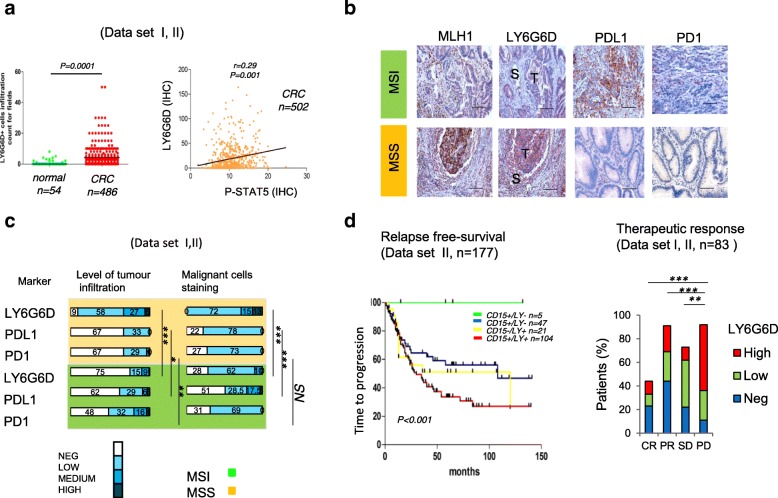
Immune inhibitory molecules in MSI and MSS tumours. **a** quantification of infiltrating LY6G6D positive cells expressed as mean of five replicates counts, cells mm^− 2^) in normal mucosa and CRC samples. Correlation between LY6G6D+ cells, p-STAT5 staining in CRC samples (five replicates counts, cells mm^− 2^). **b** Examples of MSI and MSS CRC stained by immunohistochemistry against MLH1, LY6G6D, PDL1 and PD1. T, Tumour, S, stromal compartment. Scale bar, 50 μm. **c** Quantification of stromal infiltration and staining of malignant cells by immunohistochemistry for LY6G6D, PDL1 and PD1. P* < 0.05, P** < 0.01, ***P < 0.001, by the Chi-squared test. **d** Kaplan–Meier curve showing the time to disease progression in relation to LY6G6D and CD15 status (*n* = 187); The *p-value* by log-rank test. Response to treatment according to LY6G6D IHC in primary metastatic tumors (*n* = 83) subdivided for complete (CR); partial (PR) responses; stable disease (SD) and progression disease (PD); P* < 0.05, P** < 0.01, ***P < 0.001, by the Chi-squared test

The number of infiltrating LY6G6D positive cells was significantly higher in CRC than in normal mucosa. In fact, tumours with increased number of infiltrating leucocytes exhibited stronger LY6G6D expression in malignant cells (Fig. 2c and 3a).

We next explored the nature of immune tumor microenvironment in MSI and MSS tumours by immunohistochemistry. We found that intratumoral staining of LY6G6D in both malignant and infiltrating immune cells was higher in MSS than MSI tumors (Fig. 3b and c). In contrast, MSI subset exhibited stronger PD1 and PD-L1 staining as compared to MSS tumours (Fig. 3b and c). Accordingly, JAK1 staining revealed the presence of higher levels of JAK1 in MSI than in MSS. Moreover, tumours positive for JAK1, PD1 and PDL1 were associated to a favorable prognosis as compared to those negative ones (Additional file 3: Figure S4a-d). Notably, phospho-Stat5 (P-STAT5) and LY6G6D positive staining displayed a direct correlation, so that, tumours with high levels of P-STAT5 and LY6G6D (LY6G6D^hi^) were associated with a shorter patients survival rate (Fig. 3a and Additional file 3: Figure S3d and Figure S4a-d).

Consistent with these results, the screening of dataset II alone confirmed that LY6G6D^hi^ tumours correlated with shorter disease-free survival and a reduced response to therapy compared to LY6G6D-^low/neg^ tumours (Fig. 3d and Additional file 3: Figure S4d). In this latter database, coexpression of LY6G6D^hi^/CD15^hi^ resulted in a poorer clinical outcome compared to each marker alone. We observed that the relapse-free survival at 5 years was only 33,6% for LY6G6D^hi^/CD15^hi^ patients compared to more than 95% for CD15^neg^/LY6G6D^neg^ subgroup. Intermediate results were obtained for the remaining group of patients (Fig. 3d). These results suggested that elevated levels of LY6G6D and CD15 may promote disease progression by inducing immune subversion of the tumor microenvironment.

### JAK/STAT and MEK inhibitors potently suppress the growth of poorly immunogenic CRC cells

**Fig. 4:**
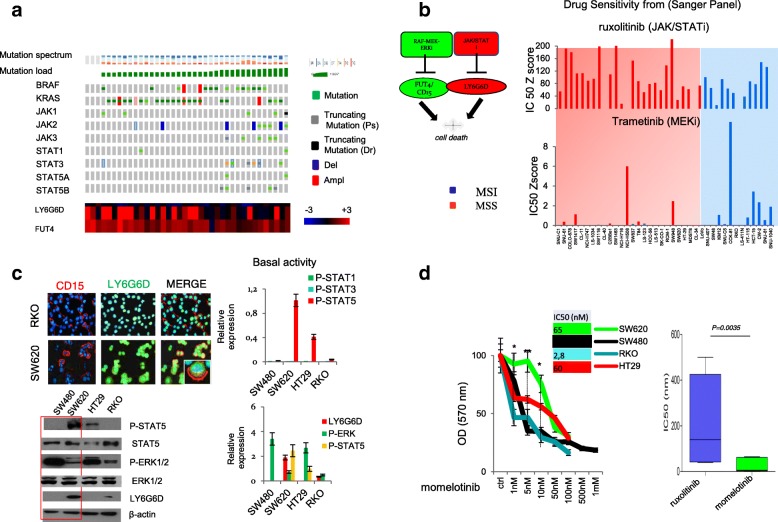
Response to JAK/STAT and MEK inhibitors in CRC molecular subtypes. **a** Heat map showing mutation/expression of JAK/STAT genes in relation to mutation load, LY6G6D and CD15/FUT4 expression in CRC cancer cell lines (*n* = 38). **b** A chemo-immune-sensitizer approach targeting LY6G6D and CD15/FUT4 by JAK/STAT and MEK inhibitors. Right, Log10 IC50 values for treatment of MSI and MSS CRC cell lines with ruxolitinib (JAK/STATi) and trametinib (MEKi) extracted from the Genomics of Drug Sensitivity in Cancer project. **c** RKO (MSI-H) and SW620 (MSS) stained with LY6G6D (green) and CD15 (red). Bottom right, basal activation of stat1, stat3, stat5 in a panel of CRC cell lines. Down left, western blotting showing expression of P-STAT5, STAT5 P-ERK1/2, ERK1/2 and LY6G6D. Down right, quantification of P-STAT5, P-ERK1/2 and LY6G6D relative to β-actin. **d** Cells were treated with different concentrations of momelotinib (range, 1 nM to 1 mM for 96 h) and evaluated for proliferation by MTT staining. Right, boxplot of log10 IC50 values for treatment of five CRC cell lines (RKO,HT29, SW480, SW620, HCT116) with ruxolitinib vs momelotinib. Results are representative of three biological replicates. *P-value* by two-tailed Student’s. P* < 0.05, **P < 0.01

To understand how ERKs and JAK/STAT5 signaling regulates *CD15/FUT4* and *LY6G6D* function, we first investigated the treatment response of human CRC cells (*N* = 38) to the MEK inhibitor, Trametinib, and the JAK1/2 inhibitor, ruxolitinib, by integrating genomic and transcriptomic data from the Cancer Cell Line Encyclopedia (CCLE) and Genomics of Drug Sensitivity from Cancer project, https://www.cancerrxgene.org/ (Fig. 4a and b). This screening revealed that in poorly immunogenic cells (MSS), characterized by the lack of JAK–STAT mutations, *CD15/FUT4* and *LY6G6D* upregulation have a low sensitivity to the JAK inhibitor. In contrast, the treatment with trametinib was highly effective in this subset of tumours (Fig. 4a and b).

To corroborate these findings, we employed in-house CRC cell lines (RKO, HCT116, HT29, SW620, SW480) to analyze *CD15/FUT4* and *LY6G6D* expression. According with previous data, CD15 was predominantly localized in the plasma membrane, while LY6G6D formed aggregates-like structure into the cytosol that were more abundant in MSS than in MSI cells (Fig. 4c). Notably, we found that ERKs and STAT5 activation under basal conditions were inversely correlated. The highest levels of P-STAT5 were detected in metastatic cell lines (i.e. SW620), which were characterized by enhanced LY6G6D protein expression (Fig. 4c).

To further characterize which JAK/STAT inhibitor potentially interfered with LY6G6D, we treated colon cancer cells with ruxolitinib or momelotinib (Mom) alone. Notably, CRC cell lines were significantly more sensitive to momelotinib than ruxolitinib (around 30-fold) (Fig. 4d and Additional file 3: Figure S5a). A screening from the cancer therapeutics response portal (https://portals.broadinstitute.org/ctrp/) to find molecule drugs that target the JAK/STAT cascade more selectively, confirmed that momemolitib was one of the most effective molecules to inhibit STAT5 signaling (Additional file 3: Figure S5b). In line with this, STAT5b copy number variations was directly correlated with the sensitivity to momemolitib in a panel of colon cancer cells (*N* = 34; pearson correlation 0.308, Additional file 3: Figure S5b). These results indicated that momelotinib could be a good candidate to inhibit STAT5/LY6G6D axis, supporting further investigation about its effects when used in combination with the MEK inhibitor.

**Fig. 5:**
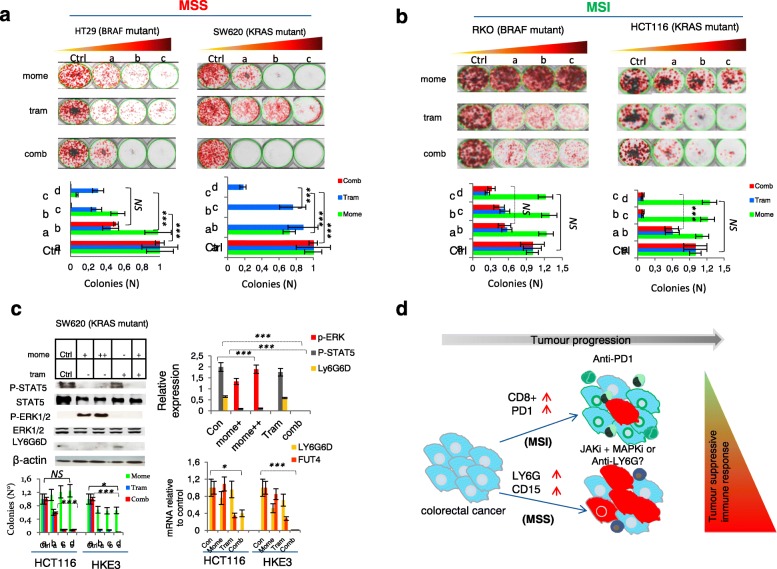
MSS CRC cell lines are highly sensitive to STAT5/MEK inhibitors. **a** MSS BRAF(V600E), KRAS mutant and **b** MSI BRAF(V600E), KRAS mutant CRC cells were seeded at low confluence and treated with increasing concentrations (lower than IC50 values) of momelotinib, trametinib or in combination (comb) twice a week. Viability was assessed by a colony formation assay. Cells were fixed, stained, and photographed after 10 days of culture. For each cell line, in the low panel the percent of cell growth inhibition determined by treatment is shown. Results represent three separate experiments, each performed in triplicate. *P-value* by two-tailed Student’s *(*related to untreated vehicle control) are shown, P* < 0.05, **P < 0.01, ***P < 0.001, NS, non significant. **c** representative immunoblot of phosphorylated STAT5 and ERK1/2 compared to LY6G6D following treatment with momelotinib, trametinib or combination. Bottom right, quantification to β-actin. Low left, viability of HCT116 cell lines (KRAS mutant), and its derivative HKE-3 KRAS wild type (KRASWT) to momelotinib, trametinib or their combination assessed by colony formation assay. Low on the right, quantification of *LY6G6D* and *FUT4* mRNA by RT-PCR analysis following drug treatments. ***P < 0.001 by Mann–Whitney *U* test. **d** Illustration of immune suppressive pathway mediated by LY6G6D and CD15, which could predict response to JAK- and MAPK-directed therapies in microsatellite stable CRC

Indeed, we found that the treatment with momelotinib or trametinib and its combination (JAKi/MEKi) resulted in a dramatic suppression of the growth rate of CRC cells known to have a primary resistance to Mab cetuximab (anti-EGFR) [24] (Additional file 3: Figure S5c and d). Consistent with these results, we extended the pharmacological treatment to a largest panel of cells analyzing colony formation. Interestingly, the JAK1/2 inhibitor enhanced the growth inhibitory effect of the MEK-inhibitor, particularly in poorly immunogenic *BRAF* or *KRAS* mutant cancer cells (HT29 and SW620) (Fig. 5a). In contrast, more immunogenic cancer cells with endogenous *JAK2* deletion and *BRAF* or *KRAS* mutation (RKO and HCT116) displayed tolerance to momelotinib, reducing the combinatory effect (Fig. 5b). Taken together, these results indicate that tumours bearing *LY6G6D* and *CD15* might be targeted by this treatment strategy, particularly those from MSS CRC subgroup.

### The JAK/STAT inhibitor, momelotinib, increases cell-death through targeting STAT5/LY6G6D axis

To understand whether the treatment with momelotinib affected the STAT5-mediated LY6G6D upregulation, we used as a metastatic model, the SW620 cell line, which is characterized by endogenous STAT5 activation and increased LY6G6D expression. We observed that momelotinib vigorously inhibited both STAT5 activation and endogenous LY6G6D expression (Fig. 5c). According with this datum, transient *STAT5* silencing markedly reduced LY6G6D levels, indicating that both pharmacologic and genetic blockade of STAT5 signaling results in *LY6G6D* downregulation (Additional file 3: Figure S6a).

Notably, momelotinib induced a robust dose-dependent activation of ERKs signaling in a heterogeneous panel of cancer cells regardless of (HER3) activation (Fig. 5c and Additional file 3: Figure S6a,b). A similar effect on ERKs activation was observed upon exposure to ruxolitinib, supporting the hypothesis that colon cancer cells became sensitized to JAK inhibitors when they are treated with inhibitors of MEK-ERK pathway [25] (Additional file 3: Figure S6b). The treatment with trametinib did not alter *LY6G6D* expression, while confirmed *FUT4/CD15* as a key mediator of RAF-MEK-ERK pathway [5] (Fig. 5c and Additional file 3: Figure S6c). Analysis of different MEKi using the public database, GDS5029 [24], supported that *STAT5/LY6G6D* signaling is a downstream mediator of resistance to MEKi in KRAS mutant CRCs (Additional file 1: Figure S6d). In addition, blocking the RAF-MEK-ERK cascade by trametinib, we found an increased phosphorylation of STAT3, another member of the Stat family (Additional file 3: Figure S6c).

Given that KRAS or JAK1/2 mutations interfere with the IFN/STAT signaling pathway [26, 27], it was tested the sensitivity to the MEK/JAKi treatment in HCT116 cell line (bearing mutant K-Ras) and its derivative HKE-3 with wild type KRAS (wtK-Ras). We observed that the cell line expressing wtK-Ras displayed a lower viability in response to the drug combination than the cell line expressing mutant K-Ras (Fig. 5c). Remarkably, the combined treatment significantly abrogated *LY6G6D* and *CD15/FUT4* expression in the cell line expressing wtK-Ras, but not in that with mutant KRAS (HCT116) (Additional file 3: Figure S6d). Therefore, these results indicate that upregulation of *LY6G6D* and *CD15/FUT4* can be efficiently abolished using a combination of JAK/STAT and MEK inhibitors in poorly immunogenic CRC subset.

## Discussion

It has been hypothesized that genetic alterations affecting signaling pathways can produce malignant variants resistant to immune effectors. We here provide evidence that LY6G6D is an antigen activated through JAK/STAT5 pathway in poorly immunogenic CRCs. Our hypothesis is also consistent with previous studies where systemic Ly6G+ cell depletion suppresses colitis-associated tumorigenesis and ApcMin/+ adenoma formation through CXCR2-dependent tumor-associated leukocytes [28]. Therefore, LY6G6D might also act as a critical mediator of malignant growth and immune evasion in human CRC. We have confirmed LY6G6G expression in both cancer cells and Regulatory T cells (Treg), which are a highly immune-suppressive subset of T cells that prevents the development of effective antitumor immunity [29]. Genomic data from the TCGA database are in agreement with our observations that both Tregs and myeloid-derived suppressor cells (MDSCs), are enriched in non-hypermutated tumors but not in MSI CRCs [9, 12, 13]. Consistent with this, *LY6G6D* expression is also enhanced in rare immune diseases, such as the autosomic dominant monocytopenia, characterized by systemic immune suppression.

Notably, STAT5 activation is also required for the expansion of Treg or induction of specific immune checkpoint molecules [30, 31]. Ly6 genes may inhibit the formation of membrane attack complexes in tumor cells hampering cytolytic activity of T cells. However, the identity of their interacting partners remains a mystery [16]. Our observations reveal that MMR proficient cancers characterized by less CD8+ T lymphocytes and low expression of PD-1/PD-L1 exhibit enhanced LY6G6D expression and STAT5 activation, implicating Ly6 genes as novel candidates for the development of new targeted therapies [17, 18] (Fig. 5d).

The functional activity of JAK/Stat5 pathway can be regulated by the ERKs signaling, which controls the transcriptional upregulation of FUT4, resulting in an increased cell-surface expression of CD15 and resistance to the anti-EGFR agents [5, 32]. In line with this, coexpression of LY6G6D and CD15 increases the risk of disease progression in response to therapy, suggesting that both MAPK and Stat5 pathways likely foster colon cancer progression in concert.

Although dysregulated JAK–STAT signaling represents an attractive therapeutic target for modulating the immune responses [33], JAKs inhibitors have shown limited clinical utility in solid tumours, including patients with refractory colorectal adenocarcinoma [34, 35]. A potential mechanism would be that mutations in *JAK1/JAK2* might block PD-L1 induction, protecting cancer cells from immune attack [34]. Not unexpectedly, we found that CRC cell lines mutant for JAK2 were less sensitive to the JAK/STAT inhibitor, momelotinib. Conversely, LY6G6D positive MSS cells (SW620) suppress T cell proliferation through the expansion of myeloid-derived suppressor cells, which were more vulnerable to momelotinib [36]. In this context, a recent study suggests that momelotinib reduces the number of cancer stem cells associated to tumor burden in a mouse model of human ovarian cancer [37]. However, in CRC cancer cell lines aberrant activation of MAPK signaling (RAS mutation or ERKs activation) was consistently involved in the mechanism of resistance to momelotinib.

Ruxolitinib, the first JAK/STAT inhibitor approved by the FDA, enhances the expression of angiogenic factors by inducing NK cell–mediated tumor progression, suggesting that combined targeting of JAK/STAT and VEGF signaling can improve therapeutic response [34]. This idea is supported by our observation that combined analyses of CD15 and LY6G6D should be evaluated as predictive biomarkers for the response to JAK- and MAPK-directed therapies. The anti-Ly6G antibody has been routinely used to deplete Ly6G^+^ cells in mice models, and it is well-tolerated and effective in a long term treatment. Its use might be an additional promising strategy to confer susceptibility to therapies in non-immunogenic and refractory human CRC. However, the treatment effects in human patients with colorectal cancer may significantly differ. Therefore, further investigation will be required to fully elucidate the mechanism by which LY6G6D promotes cancer progression.

## Conclusions

We here provide evidence that LY6G6D and CD15 promote chemo-immune-resistance in immunologically compromised colon cancers and can be used as biomarkers to decide patients treatment. Further preclinical studies would reveal if LY6G6D and CD15 antagonists, in addition to established chemotherapeutic protocols, can improve therapy response in refractory colorectal cancer.

## Additional file

Additional file 1: Materials and Methods and any associated references. (DOCX 45 kb)

Additional file 2: **Table S1-S2**. Clinicopathological characteristics of CRC Patients named dataset (I) investigated by tissue microarrays (TMAs) and **Table S2**. Clinicopathological characteristics of CRC Patients named (dataset II) investigated by TMAs. (DOC 74 kb)

Additional file 3: **Figures S1-S6**. (PDF 1120 kb)

## Abbreviations

*ANOVA*: Analysis of variance
*BRAF*: B-Rapidly Accelerated Fibrosarcoma
CIN: Chromosomal instability
CRC: Colorectal cancer
CTLA-4: Cytotoxic T-Lymphocyte Antigen 4
EGFR: Epidermal growth factor receptor
*ERK*: Extracellular-Signal Regulated Kinase
*FDA*: Food and Drug Administration
*FUT4*: Fucosyltransferase 4
*GEO*: Gene Expression Omnibus
*GSEA*: Gene Set Enrichment Analysis
*IHC*: Immunohistochemistry
JAK: Janus kinase
KRAS: Kirsten Rat Sarcoma viral oncogene homolog
LY6G6D: Lymphocyte antigen 6 complex, locus G6D
*MAPK*: Mitogen-Activated Protein Kinase
MSS: Microsatellite-stable
PD1: Programmed cell death protein
*PIK3CA*: Phosphoinositide 3-kinase
*qRT-PCR*: Quantitative real time polymerase chain reaction
STAT: Signal transducer and activator of transcription
*TCGA*: The Cancer Genome Atlas
Th: T helper
Treg: Regulatory T cells
