# Supplementary material for: JAK/Stat5-mediated subtype-specific lymphocyte antigen 6 complex, locus G6D (LY6G6D) expression drives mismatch repair proficient colorectal cancer
Source: J Exp Clin Cancer Res. 2019 Jan 22;38:28. doi: 10.1186/s13046-018-1019-5 (PMC6343337; doi:10.1186/s13046-018-1019-5)
Supplement: Supplementary file 1 — Materials and Methods and any associated references. (DOCX 45 kb) [file 13046_2018_1019_MOESM1_ESM.docx]

**JAK/Stat5-mediated subtype-specific Lymphocyte Antigen 6 Complex, Locus G6D (LY6G6D) expression drives mismatch repair proficient colorectal cancer**

Guido Giordano^1,2†^, Pietro Parcesepe^3†^, Mario Rosario D’Andrea^2^, Luigi Coppola^2^, Tania Di Raimo^2^, Andrea Remo^4^, Erminia Manfrin^4^, Claudia Fiorini^3^, Aldo Scarpa^3^,Carla Azzurra Amoreo^5^, Fabiana Conciatori^6^, Michele Milella^6^, Francesca Pia Caruso^7,8^, Luigi Cerulo^7,8^_,_ Almudena Porras^9,10*^ and Massimo Pancione^7,9*^.

^1^ Department of Oncology, Casa Sollievo della Sofferenza-IRCCS, San Giovanni Rotondo, Italy; ^2^Medical Oncology and Anatomic Pathology Unit, San Filippo Neri Hospital, Rome, Italy;^3^Department of Diagnostics and Public Health – Section of Pathology, University and Hospital Trust of Verona, Verona, Italy; ^4^Pathology Unit, “Mater Salutis” Hospital AULSS9, Legnago (Verona), Italy; ^5^Pathology, IRCCS Regina Elena National Cancer Institute, Rome Italy, Via Elio Chianesi 53, 00144, Rome, Italy, ^6^Medical Oncology, IRCCS Regina Elena National Cancer Institute, Rome Italy, Via Elio Chianesi 53, 00144, Rome, Italy; ^7^Department of Sciences and Technologies, University of Sannio, Benevento, Italy; ^8^Bioinformatics Laboratory, BIOGEM scrl, Ariano Irpino, Avellino, Italy; ^9^Department of Biochemistry and Molecular Biology, Faculty of Pharmacy, Complutense University Madrid, Spain;^10^Health Research Institute of the Hospital Clínico San Carlos (IdISSC), Madrid, Spain.

**MATERIALS AND METHODS**

Materials and Methods and any associated references are described more in detail within the supplementary material.

**Gene expression data analysis among different tumor subtypes**

The Gene Expression Profile from 604 cancer cell lines representative of 14 different tumor sites from the Cancer Cell Lines Encyclopedia series were analyzed. We selected a collection of ~6,000 known human genes with immunomodulatory functions from InnateDB, Innate Immunity Genes curated database (<http://allergen.innatedb.com/>). ANOVA analysis was adopted to test for differential expression among different tumor subtypes and eta squared was used to determine those with a greater effect size. A series of 55 colorectal cancer cell lines from the Cancer Cell Lines Encyclopedia was selected to visualize DNA copy number and mutational load. A total of 17 cancer types: Colorectal (n=597); Glioma (n=153), Thyroid (n=501); Lung (n=994); Liver (n=365); Pancreas (n=176); Head/Neck (n=499); Stomach (n=354); Urothelial (n=406); Renal (n=877); Prostate (n=494); Testis (n=134); Breast (n=1075); Cervical (n=291); Ovarian (n=373); Endometrial (n=541); Melanoma (n=102) reported as median FPKM (number Fragments Per Kilobase of exon per Million reads), were retrieved from The Cancer Genome Atlas ([TCGA](https://cancergenome.nih.gov/)) dataset to analyze gene expression levels.

**Immune cell type enrichment analysis**

To analyze the expression changes related to specific immune cell subpopulations, we applied a deconvolution approach based on Gene Set Enrichment Analysis (GSEA). Unsupervised hierarchical clustering was then applied on the Euclidean distance and Ward linkage method on the matrix of the enrichment scores.

**Patients samples and tissue microarrays analysis**

Two independent datasets of patients with sporadic CRC were retrospectively recruited and collected **(supplementary Tables 1 and 2).** Additional samples included snap**-**frozen liquid nitrogen biopsies from tumor and matched normal adjacent mucosa. The recruitment of the patients was performed in accordance with the ethical guidelines, protocol number: 1703/2016 of September 2016 from the San Filippo Neri Hospital, Rome, Italy. The tissue microarrays (TMAs) used for this study included tumour tissues from 516 unselected colon carcinoma and 92 corresponding normal mucosa specimens. The TMAs were processed as previously described in detail^55^. Briefly, the corresponding area on the matching formalin-fixed, paraffin-embedded tissue (donor block) was then identified and marked. Tissue cylinders with a 2 mm diameter were punched from representative tissue areas of each donor tissue block and placed into one recipient paraffin blocker. Each TMA spot included at least 50% tumor cells.

**Immunohistochemistry**, **immunofluorescence and western blot on tumor tissues**

Immunohistochemistry (IHC) was performed using 4-μm-thick histological TMA sections. The antibodies used in this study were the following: anti-LY6G6D (ab139649 Abcam, Cambridge, UK); Anti-STAT5 (phospho Tyrosine 694, Abcam, Cambridge, UK), anti-JAK1 (ab47435 Abcam, Cambridge, UK); Anti-PD1 antibody (ab137132 Abcam, Cambridge, UK); anti-PD-L1 (ab205921, Abcam, Cambridge, UK), anti-MSH2 (ab92372, Abcam, Cambridge, UK), anti-MLH1 (ab92312, Abcam, Cambridge, UK), anti-MSH6 (ab92471, Abcam,Cambridge, UK) and anti-CD8 (ab4055, Abcam, Cambridge, UK), anti-CD15 antibody [FUT4/1478R] (ab218403, Abcam,Cambridge, UK).

TMA slides were stained individually with horseradish peroxidase-conjugated avidin biotin complex (ABC) as previously reported for immunohistochemistry ^5.^ Infiltrating immune cells were counted automatically by using ImageJ-based software. All the cell counts were expressed as cells mm^-2^. The proportion of cancer cells stained was scored regardless of intensity as follows:1) Negative staining (Neg) was defined as the complete absence of staining in more than 95% of tumor cells; 2) Low expression characterized by a limited number of tumor cells scattered in a background of either negative or positive tumor cells. 3) High expression corresponded to a homogeneous membrane staining in virtually all tumor cells.

For double immunofluorescence, 4-μm-thick histological sections were obtained from whole paraffin embedded blocks and deparaffinised in graduated alcohol as reported ^51^. Pretreatment occurred in a water bath for 40 minutes at 90°C in 0.1M sodium citrate (pH 7.2). The slides were blocked for 20 minutes at room temperature with 20% BSA and then incubated at 4°C overnight with mixtures of two primary antibodies; anti-LY6G6D (ab139649 Abcam, Cambridge, UK); anti-CD8 (ab4055, Abcam, Cambridge, UK), anti-CD4 antibody (ab133616, Abcam,Cambridge, UK) anti-FOXP3 (NB100-39002) Novus Biological. The optimal dilution of each antibody was established before by single staining immunofluorescence staining. After overnight incubation, slides were washed in PBS once and incubated in the dark for 1 hour at 37°C with secondary antibodies specific for the appropriate species, conjugated with Cy3, and an anti-rabbit antibody, conjugated with FITC, were diluted 1:500, 1:1000 in PBS respectively. Slides were then again washed in PBS and mounted in Vectashield Mounting Medium with DAPI and examined with an Olympus IX81 deconvolution fluorescence microscope (Olympus Microscopes, Center Valley, PA).

For western blot analysis, tissues were lysates in a buffer containing 50 mM Tris·HCl (pH 7.5), 150 mM NaCl, 1% NP40, 5 mM EGTA, 5 mM EDTA, 1 mM phenylmethylsulfonyl fluoride, 10 μg/ml aprotinin, 10 μg/ml leupeptin, 1 mM Na_3_VO_4_ and 20 mM NaF and centrifuged (at 13.000 rpm 10 min, 4°C). Western-blot analysis was carried out as previously described using total tissue extracts from the tumor and adjacent normal mucosa ^54^. Proteins were separated by electrophoresis using SDS-page gels and transferred to nitrocellulose membranes that were probed with the following antibodies against: anti-LY6G6D (ab139649 Abcam, Cambridge, UK); anti-CD15 antibody [FUT4/1478R] (ab218403, Abcam,Cambridge, UK), (β-actin, dilution 1:10000, Sigma Aldrich) was used as loading control.

**Cell Lines**.

Human colon cancer cell lines HCT116, HT29, RKO, SW480 and SW620 were purchased from American Type Culture Collection (ATCC, Rockville, MD, USA) and cultured as appropriate at 37 °C in 5 % CO2 atmosphere in Dulbecco’s modified Eagle’s medium, DMEM (Thermo fisher scientific, Whaltam, MA, USA) or RPMI 1640, (Thermo fisher scientific, Whaltam, MA, USA) supplemented with 10 % fetal bovine serum (FBS) or when required without FBS, 100 U/ml penicillin and 100 ng/ml streptomycin (Thermo fisher scientific, Whaltam, MA, USA). The human HCT116 (KRAS mutant), and its derivative HKE-3 KRAS wild type (KRASWT) cell lines were kindly provided by Dr. M. Milella (Regina Elena National Cancer Institute, Rome, Italy).The genotype of parental cell lines were confirmed by short tandem repeat (STR) genotyping. The STR genotypes were consistent with published genotypes for each cell lines. All cell lines were found to be free of mycoplasma tested with the mycoAlert Mycoplasma Detection Kit (Lonza, Basel, Switzerland).

**Drugs**

Cetuximab, an anti-EGFR human-mouse chimeric monoclonal antibody (MAb) was kindly provided by Dr. Guido Giordano, (Department of Oncology, Casa Sollievo della Sofferenza-IRCCS, San Giovanni Rotondo, Italy).Trametinib, a selective MEK1/2 tyrosine kinase inhibitor, was kindly provided by Dr. M. Milella (Regina Elena National Cancer Institute, Rome, Italy). Ruxolitinib and Momelotinib, two selective JAK/STAT inhibitors were kindly provided by Dr. Guido Giordano, (Department of Oncology, Casa Sollievo della Sofferenza-IRCCS, San Giovanni Rotondo, Italy). All inhibitors were dissolved in sterile dimethylsulfoxide (DMSO) and a 10 mM working solution was prepared and stored in aliquots at -20°C. Working concentrations were diluted in culture medium just before each experiment. We mined the data from the Genomics of Drug Sensitivity in Cancer project (Sanger panel) to see the sensitivity of colon cancer cell lines MEKi and JAK/STATi drugs^56^. Data were available for more of 30 cancer cell lines that were tested for sensitivity to both trametinib and ruxolitinib. Sensitivity to other MEKi tested on CRC cell lines was analyzed by using independent database such as the GDS5029 ^5^.

A set of 481 small-molecule probes that collectively modulate a broad array of cell processes (<https://portals.broadinstitute.org/ctrp/>) was used to identify cancer dependencies to JAK/STAT inhibitor molecules.

**Proliferation Assay.**

Cancer cell lines were seeded in 24-well plates and were treated with different concentrations of drugs alone or in combination: cetuximab, Trametinib (range, 0.5 nM to 1uM), ruxolitinib or momelotinib (range, 1nM to 1uM) for 96 hours. Cell proliferation was measured with the 3-(4,5-dimethylthiazol-2-yl)-2,5- diphenyltetrazolium bromide (MTT). The IC50 was determined by interpolation from the doseresponse curves. Results represent the median of three separate experiments, each performed in quadruplicate. Results of the combination treatment were analysed according to the method of Chou and Talalay by using the CalcuSyn software programme (Biosoft, Cambridge, UK).

**Long-Term Proliferation Assays**

For momelotinib and trametinib treatments, cells were seeded into six-well plates (5 x 10^4^ cells/well;) and cultured for 10 days both in the absence and presence of drugs as indicated. The different concentrations of drugs alone or in combination were determined to interpolation dose response curves ≤IC50 values. At the endpoints of colony formation assays, cells were fixed, stained with crystal violet, and photographed. All relevant assays were performed independently at least three times.

**Immunofluorescence and Immunoblotting analysis**

About 100,000 cells were plated on coverslips; after 24 h, they were washed twice in phosphate-buffered saline and fixed with 4 % paraformaldehyde in phosphate-buffered saline for 10 min at room temperature. Blocking was performed with 3 % bovine serum albumin, and the primary antibodies anti-LY6G6D diluted 1:50; anti-CD15 diluted 1:100 were incubated for 30 min at room temperature. Fluorescently conjugated secondary antibody with Cy3 or FITC, were diluted 1:1000 in PBS respectively. Slides were then again washed in PBS and mounted in Vectashield Mounting Medium with DAPI and examined with an Olympus IX81 deconvolution fluorescence microscope (Olympus Microscopes, Center Valley, PA). For western blot analysis, cells were seeded in 6-well plates and treated with vehicle, drug alone or their combination for 24 hours. Protein lysates containing comparable amounts of proteins, estimated by a modified Bradford assay (Bio-Rad, Munich, Germany), were subjected to Western blot, as previously described^55^. Immuno-complexes were detected with the enhanced chemiluminescence kit (Pierce Biotechnology Inc, Rockford, USA). The flowing primary antibodies were used: Anti-ERBB3 Antibody (1455, Biocompare), Anti-STAT5 (phospho Tyrosine 694, Abcam, Cambridge, UK), Anti-STAT5 antibody (ab126832); Phospho-(Erk1/2) (Thr202/Tyr204) #9101 cell signaling, total (Erk1/2) Antibody #9102 Cell signaling; (STAT3 total, Y701-STAT3, p-STAT3, dilution 1:500, Cell Signaling Technology), anti-AKT polyclonal antibody (#9272), pAKT monoclonal antibody (#4060), from Cell Signaling (Beverly, MA, USA), β-actin, (Sigma Aldrich). A ratio of normalized ERK1/2 (pERK/total ERK1/2), Stat3,stat5 (pStat3/total Stat3; pStat5/total Stat5**)** was calculated for monitoring expression and phosphorylation levels.

**RNA interference and PCR analysis.**

The small inhibitor RNAs (siRNA) against Stat5 siRNA (h), sc-29495 were from SANTA CRUZ BIOTECHNOLOGY, INC. The siCONTROL Non-targeting Pool (Control siRNAs sc-37007, was used as a negative (scrambled) control. Cells were transfected with 50 and 100 nM siRNAs using Hiperfect reagent (Qiagen, Monza, Italy) following manufacturer’s instructions. The day before transfection, the cells were plated in 6-well plates at 40% of confluence in medium supplemented with 5% FBS without antibiotics. Cells were harvested at different time points (24, 48, 72 and 96 hours) after transfection. Western blot analysis for pSTAT5 and total STAT5 expression was done for monitoring gene expression knockdown along with other markers as previously described. After drug treatment, total RNA was isolated using RNeasy Kit (Qiagen, Hilden, DE) according manufactures’ instructions. Reverse transcription was performed using the first-strand cDNA synthesis kit (General Electric, Buckinghamshire, UK). qPCR was performed in 96-well plates using pre-designed TaqMan probe/primers on a ABI 7900HT system (Life technology, Carlsbad, CA, USA). The relative amount of mRNA was calculated using the comparative Ct method after normalization to GAPDH or rRNA 18S expression. Fast SYBR Green Master Mix from Life Technologies and 10 μM for each primer pair were used. qPCR reactions were performed on an Applied Biosystems 7500 Real-Time PCR machine using the standard amplification protocol. The primers for RT-PCR are as follows: LY6G6DFw: ATGAAACCCCAGTTTGTTGGG; LY6G6DRw CTATCCGCTCCACAGTCCTGG. For FUT4 and RNA 18S the primers were as reported ^5^.

**Statistical analysis**.

Differences in overall survival and time to disease progression was measured by Kaplan-Meier curves using the log-rank test, at 95% confidence intervals (CI). The Spearman rank test was used to assess the correlation between gene expression levels. Representation with boxplot was used to test statistical significance with student t-test (2-tailed) or Wilcoxon-Mann-Whitney tests with median differences at 95% confidence interval (CI). Data are presented with mean, medians and ranges. The statistical analyses of *in vitro* and *in vivo* data were carried out using Prism version 4.02 (GraphPad Software, Inc), GeneSpring R/bioconductor v.12.5 and R based package. All p values represent two-sided tests of statistical significance with p value < 0.05.

**References**

1. Barretina, J. *et al.* The Cancer Cell Line Encyclopedia enables predictive modelling of anticancer drug sensitivity. *Nature* 483, 603-607 (2012).
2. [Johnson WE](https://www.ncbi.nlm.nih.gov/pubmed/?term=Johnson%20WE%5BAuthor%5D&cauthor=true&cauthor_uid=16632515), [Li C](https://www.ncbi.nlm.nih.gov/pubmed/?term=Li%20C%5BAuthor%5D&cauthor=true&cauthor_uid=16632515), [Rabinovic A](https://www.ncbi.nlm.nih.gov/pubmed/?term=Rabinovic%20A%5BAuthor%5D&cauthor=true&cauthor_uid=16632515). Adjusting batch effects in microarray expression data using empirical Bayes methods. [Biostatistics.](https://www.ncbi.nlm.nih.gov/pubmed/16632515) 2007 Jan;8(1):118-27.
3. Alexandrov, L. B. *et al.* Signatures of mutational processes in human cancer. *Nature* **500**, 415-421 (2013).
4. Cancer Genome Atlas, N. Comprehensive molecular characterization of human colon and rectal cancer. *Nature* **487**, 330-337 (2012).
5. Kandoth, C. *et al.* Mutational landscape and significance across 12 major cancer types. *Nature* **502**, 333-339 (2013).
6. Hoadley, K.A. *et al.* Multiplatform analysis of 12 cancer types reveals molecular classification within and across tissues of origin. *Cell* **158**, 929–944 (2014).
7. Guinney J, Dienstmann R, Wang X, de Reynies A, Schlicker A, Soneson C et al. The consensus molecular subtypes of colorectal cancer. Nat Med 2015; 21: 1350–1356.
8. Sheffer, M. *et al.* Association of survival and disease progression with chromosomal instability: a genomic exploration of colorectal cancer. *Proc Natl Acad Sci U S A* **106**, 7131-7136 (2009).
9. Watanabe, T. *et al.* Chromosomal instability (CIN) phenotype, CIN high or CIN low, predicts survival for colorectal cancer. *J Clin Oncol* **30**, 2256-2264 (2012).
10. Smith JJ, Deane NG, Wu F, et al. Experimentally derived metastasis gene expression profile predicts recurrence and death in patients with colon cancer. Gastroenterology.2010;138:958–68.
11. Bindea, G., Mlecnik, B., Tosolini, M., Kirilovsky, A., Waldner, M., Obenauf, A. C.& Bruneval, P. (2013). Spatiotemporal dynamics of intratumoral immune cells reveal the immune landscape in human cancer. Immunity, 39(4), 782-795.
12. Subramanian, A., Tamayo, P., Mootha, V. K., Mukherjee, S., Ebert, B. L., Gillette, M. A., ... & Mesirov, J. P. (2005). Gene set enrichment analysis: a knowledge-based approach for interpreting genome-wide expression profiles. *Proceedings of the National Academy of Sciences*, *102*(43), 15545-15550.
13. Skrzypczak, M. *et al.* Modeling oncogenic signaling in colon tumors by multidirectional analyses of microarray data directed for maximization of analytical reliability. *PLoS One* **5** (2010).
14. Remo A, Manfrin E, Parcesepe P et al. [Centrosome Linker-induced Tetraploid Segregation Errors Link Rhabdoid Phenotypes and Lethal Colorectal Cancers.](https://www.ncbi.nlm.nih.gov/pubmed/29784668) Mol Cancer Res. 2018 May 21.
15. Votino C et al. [Aberrant BLM cytoplasmic expression associates with DNA damage stress and hypersensitivity to DNA-damaging agents in colorectal cancer.](https://www.ncbi.nlm.nih.gov/pubmed/27169843) J Gastroenterol. 2017 Mar;52(3):327-340.
16. Pagnotta, S. M. *et al.* Ensemble of gene signatures identifies novel biomarkers in colorectal cancer activated through PPARgamma and TNFalpha signaling. *PLoS One* **8**, e72638 (2013).
17. Garnett et al. TI - Systematic identification of genomic markers of drug sensitivity in cancer cells
    .Nature 2012
