# Supplementary material for: JAK/Stat5-mediated subtype-specific lymphocyte antigen 6 complex, locus G6D (LY6G6D) expression drives mismatch repair proficient colorectal cancer
Source: J Exp Clin Cancer Res. 2019 Jan 22;38:28. doi: 10.1186/s13046-018-1019-5 (PMC6343337; doi:10.1186/s13046-018-1019-5)
Supplement: Supplementary file 2 — Table S1-S2. Clinicopathological characteristics of CRC Patients named dataset (I) investigated by tissue microarrays (TMAs) and Table S2. Clinicopathological characteristics of CRC Patients named (dataset II) investigated by TMAs. (DOC 74 kb) [file 13046_2018_1019_MOESM2_ESM.doc]

**Table S1 .** Clinicopathological characteristics of CRC Patients named dataset (I) investigated by tissue microarrays (TMAs)

| **Characteristics** | **No.** |
| --- | --- |
| **No. of Patients** | **330** |
| **AGE**  Median | 72 |
| **STAGE AT DIAGNOSIS**  I  II  III  IV | 45  102  128  55 |
| **PRIMARY TUMOR SITE**  Right  Left | 108  222 |
| **T1**  **T2**  **T3**  **T4** | 19  37  251  23 |
| **N0**  **N1**  **N2** | 202  82  46 |
| **M0**  **M1** | 284  46 |
| **G1**  **G2**  **G3** | 12  244  74 |
| **K Ras**  Wild Type  Mutant  Unavailable | 70  31  229 |
| MMR deficient  MMR proficient  Unavailable | 52  253  25 |

**Abbreviations:** Right includes: caecum and ascending colon. Left includes: Transverse, descending colon, sigmoid and rectum. Tumor, node, metastasis (TNM) staging system, 7th edition, 2010 (AJCC, 2010). Tumor grade (G) whereby G1 is well, G2 moderately, and G3 poorly differentiated, respectively. Mismatch repair (MMR) deficient tumors showed absence of nuclear staining in at least one of following marker: MLH1 or MSH2 or MSH6 or PMS2. MMR-proficient tumors are those simultaneously expressing all these markers.

**Table S2.** Clinicopathological characteristics of CRC Patients named (dataset II) investigated by TMAs.

| **Characteristics** | **No.** |
| --- | --- |
| **No. of Patients** | **186** |
| **AGE**  Median | 69 |
| **STAGE AT DIAGNOSIS**  I  II  III  IV | 14  62  66  43 |
| **PRIMARY TUMOR SITE**  Right  Transverse  Left  Sigmoid  Rectum | 59  12  22  36  49 |
| **T1**  **T2**  **T3**  **T4** | 1  20  121  44 |
| **N0**  **N1**  **N2** | 88  56  40 |
| **M0**  **M1** | 134  52 |
| **G1**  **G2**  **G3** | 2  146  38 |
| **K Ras**  Wild Type  Mutant  Unavailable | 37  41  108 |
| MMR deficient  MMR proficient  Unavailable | 33  150  3 |
| Recurrence after surgery  No  Yes  Unavailable | 93  90  3 |

**Abbreviations:** Right includes: caecum and ascending colon. Left includes: Distal descending colon; Tumor, node, metastasis (TNM) staging system, 7th edition, 2010 (AJCC, 2010). Tumor grade (G) whereby G1 is well, G2 moderately, and G3 poorly differentiated, respectively. Mismatch repair (MMR) deficient tumors showed absence of nuclear staining in at least one of following marker: MLH1 or MSH2 or MSH6 or PMS2. MMR-proficient tumors are those simultaneously expressing all these markers.
