# Supplementary material for: JAK/Stat5-mediated subtype-specific lymphocyte antigen 6 complex, locus G6D (LY6G6D) expression drives mismatch repair proficient colorectal cancer
Source: J Exp Clin Cancer Res. 2019 Jan 22;38:28. doi: 10.1186/s13046-018-1019-5 (PMC6343337; doi:10.1186/s13046-018-1019-5)
Supplement: Supplementary file 3 — Figures S1-S6. (PDF 1120 kb) [file 13046_2018_1019_MOESM3_ESM.pdf]

Supplementary Figure 1

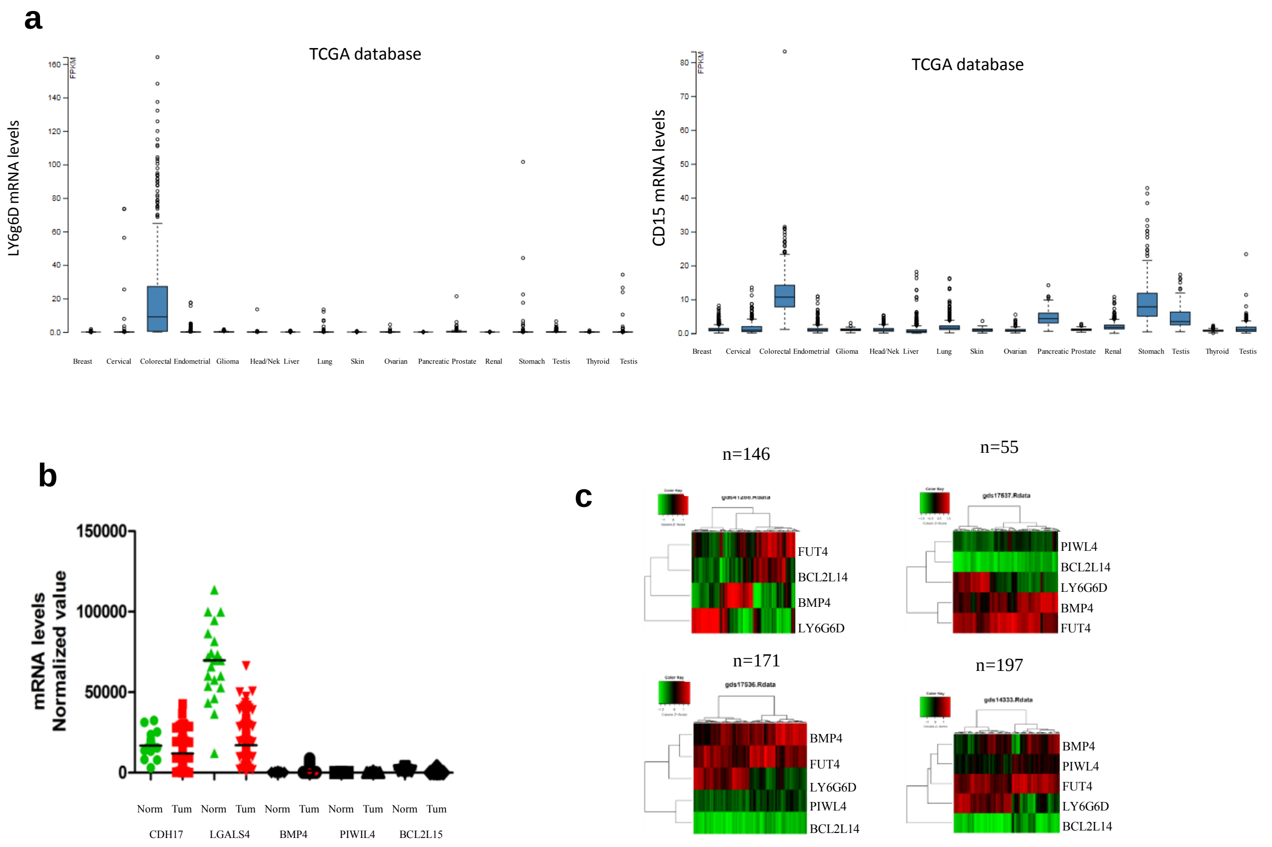

Fig. S1 Characterization of *LY6G6D* antigen in Colorectal cancer. a Relative mRNA expression levels of *LY6G6D* across *TCGA* data base comprising: Colorectal (n=597); Glioma (n=153), Thyroid (n=501); Lung (n=994); Liver (n=365); Pancreas (n=176); Head/Neck (n=499); Stomach (n=354); Urothelial (n=406); Renal (n=877); Prostate (n=494); Testis (n=134); Breast (n=1075); Cervical (n=291); Ovarian (n=373); Endometrial (n=541); Melanoma (n=102). Median, edges of the box with 25th and 75th percentiles and extremes are represented. *b* quantification of mRNA expression levels in CRC (n=210) and normal mucosa (n=20) from TCGA for a subset of immune-related genes enriched in colorectal cancer. c Heat map of differentially expressed genes deriving from the immune signature illustrating that CRCs from different datasets can be splitted in LY6G6D-low and high as compared to other genes. Red denotes high mRNA expression; green denotes low mRNA expression.

Supplementary Figure2

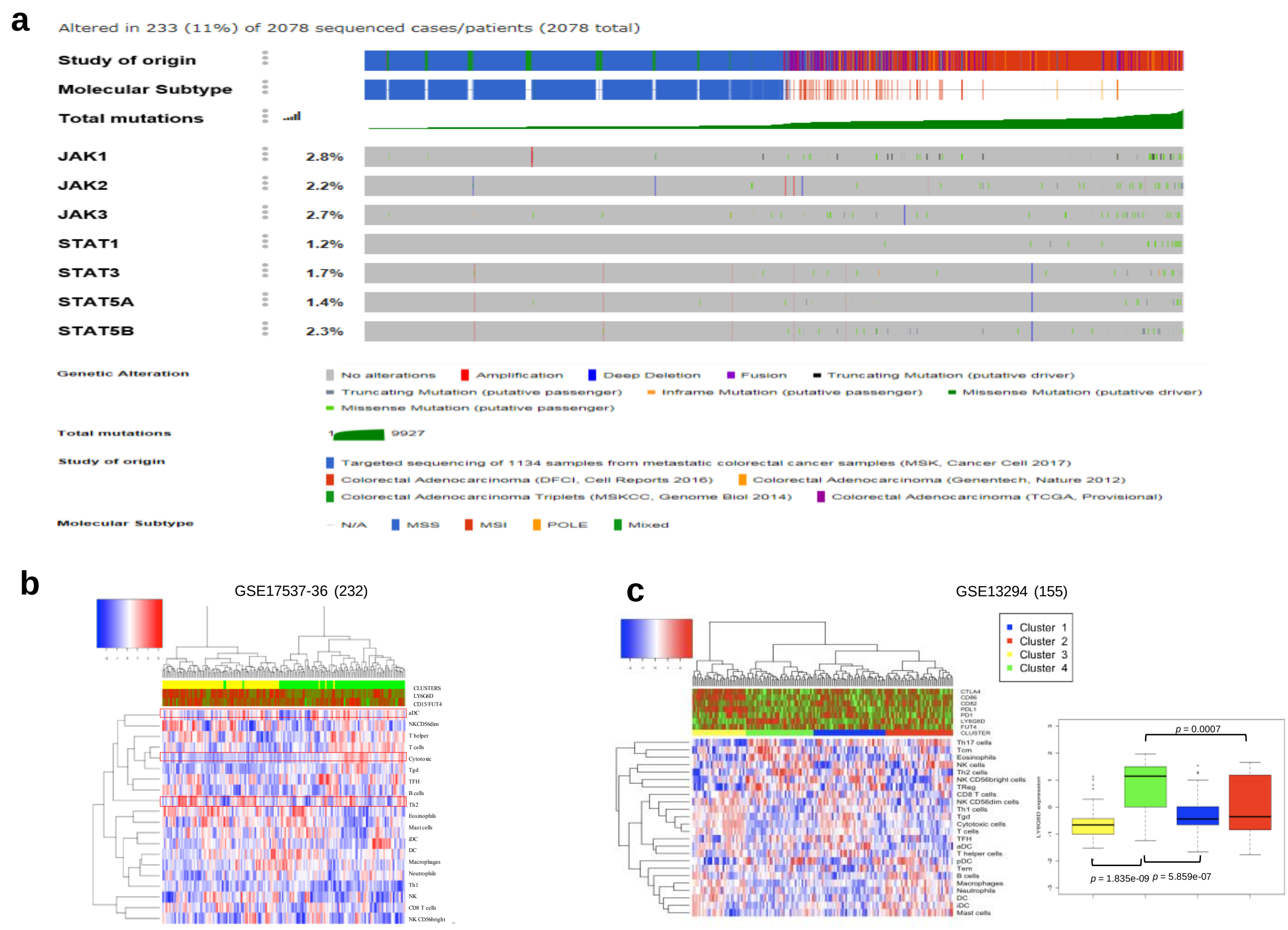

Fig. S2 JAK/STAT signaling and immune phenotypes in CRC. a Recurrence of somatic mutations in JAK/*STAT* components across multiple datasets of colorectal cancer (n=2078) stratified according to the study, mutation load and mismatch repair alterations. **b** Heatmap showing the relative abundance of immune populations estimated among 2 transcriptomic cohorts of 232 CRC patients, that were related to LY6G6D and CD15/FUT4 expression. Red denotes a higher expression and blue and green a lower expression.**c** On the left, unsupervised hierarchical cluster of 155 CRC samples (dataset: GSE13294) using cell-specific immune-signatures categorized patients into four groups, with distinct cell immune associated gene expression. Data are obtained using the Euclidean distance and Ward linkage method on the matrix of the enrichment scores calculated through ssGSEA. Right tracks represents the expression profile of known immune inhibitory molecules, together with *LY6G6D* and *CD15/FUT4* genes. On the bottom, boxplots of *LY6G6D* gene expression in each cluster.

Supplementary Figure 3

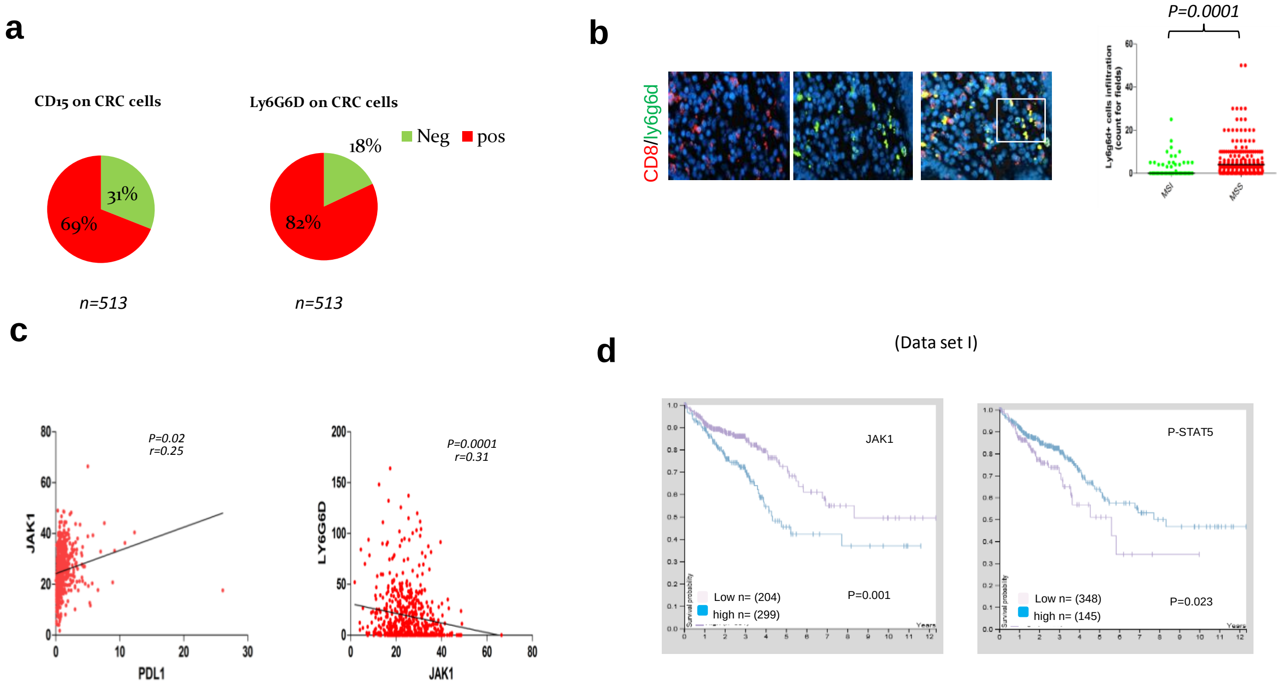

Fig. S3. *LY6G6D* and CD15/FUT4 antigens in relation to JAK/STAT in colorectal cancer specimens. a Percentage of CRCs expressing *LY6G6D* and CD15/FUT4 antigens by immunohistochemistry. **b** double immunofluorescence analysis in paraffin embedded CRC sections indicating that LY6G6D infiltrating cells do not co-localize with CD8+ T lymphocytes. **c** left, direct relation between PDL1 and JAK1 and, right inverse relationship between LY6G6D and JAK1 in CRC specimens. **d** Kaplan-Meier survival curves indicating opposite prognostic significance for JAK1 and STAT5 expression in CRC, respectively. The *P values* by log rank test.

Supplementary Figure 4

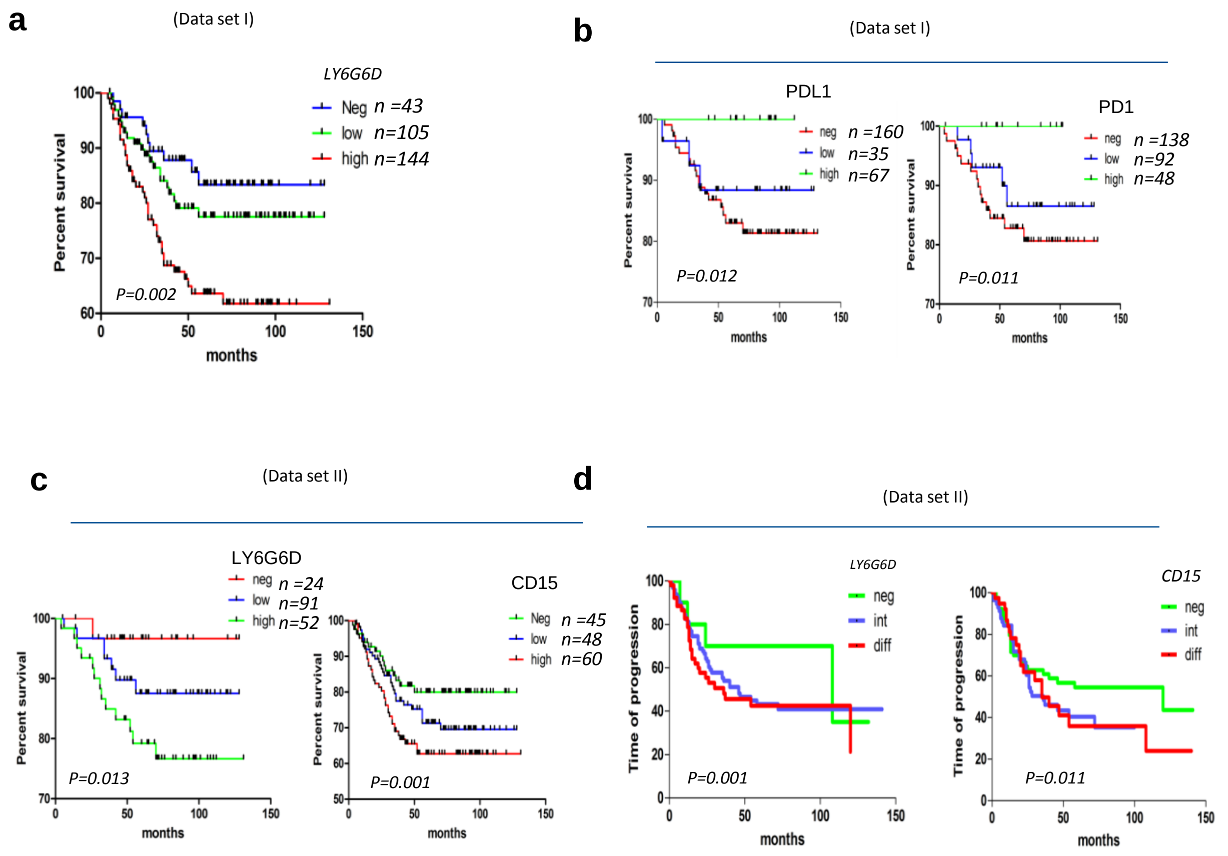

Figure S4. Prognostic significance of immune inhibitor molecules in CRC . **a** overall survival analysis in relation to *LY6G6D* IHC in CRC specimens. **b** The Kaplan-Meier overall survival analysis for PDL1 and PD1 is shown. **c** high *LY6G6D* and CD15/FUT4 expression is associated with short overall survival also in dataset II. **d** A similar trend is observed in relation to disease progression. The P values by log rank test.

Supplementary Figure 5

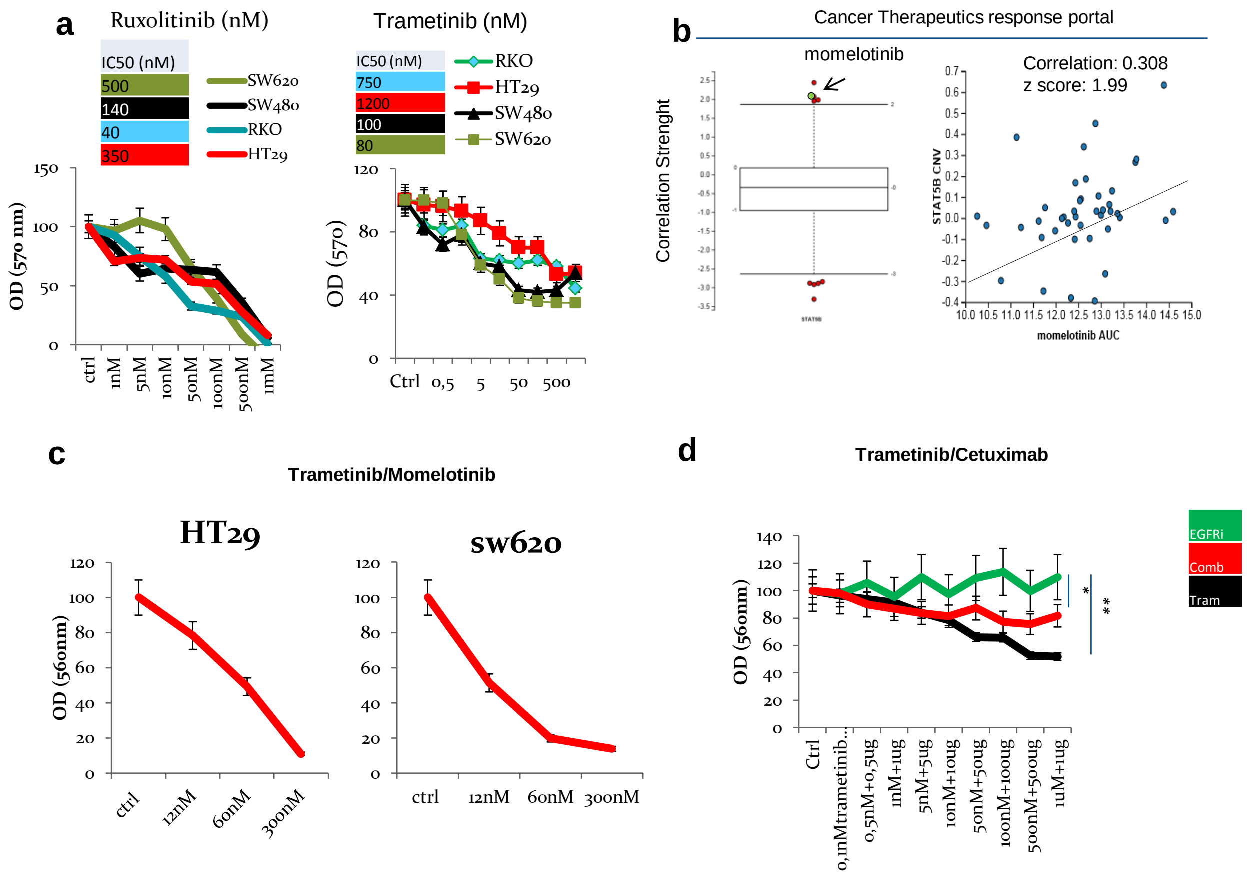

Figure S5. EGFR-unresponsive CRC cell lines are highly sensitive to trametinib and moletotinib combination. a The indicated CRC cell lines were treated every day with different concentrations of ruxolitinib or trametinib for 96 hours and cell proliferation was evaluated by MTT assay. **b** Sensitivity of characterized CRC cells to an Informer Set of small molecules that best target STAT5 nodes. Left, the JAK/STATi momelotinib results among the most effective in targeting STAT5. Right, cancer-genomic alterations at STAT5 locus copy number alterations show a close dependency to momelotinib in CRC cell lines (https://portals.broadinstitute.org/ctrp/). **c** HT29 and SW620 with primary resistance to cetuximab, were treated every day with different concentrations of trametinib and momelotinib for 96 hours at a fixed drug ratio of 1:1 and cell proliferation was evaluated by MTT assay. The same CRC cell lines were exposed to the trametinib, cetuximab or with their combination at fixed drug ratio of 1:1. *$P<0.05$ **$P<0.01$ by student *t* test (three independent experiments).

Supplementary Figure 6

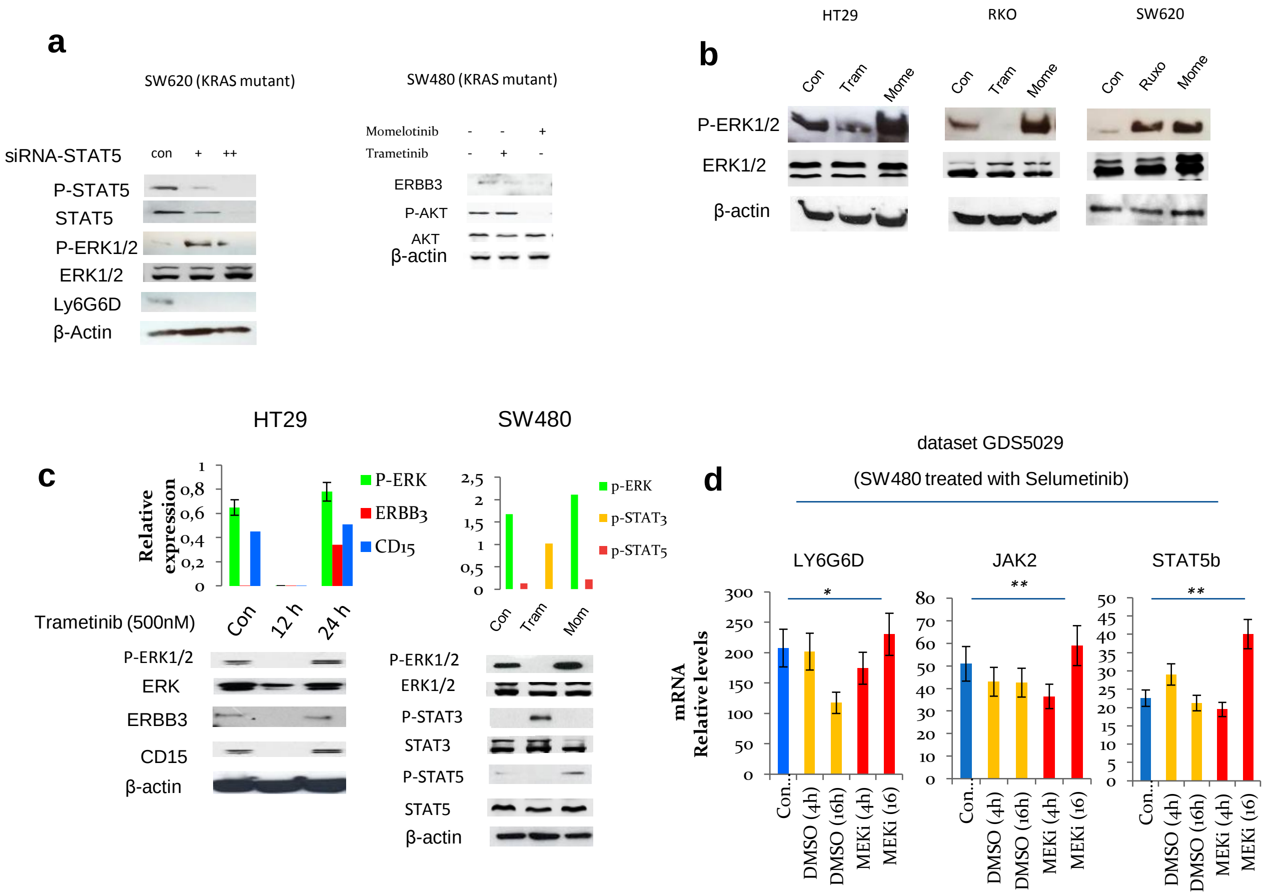

Figure S6. Resistance to JAK/STAT inhibitors mediated by activation of ERK signaling . a SW620 cells were transfected with either specific siRNA targeting STAT5 or with a control (scrambled) and harvested at 24 and 48 hours after transfection. Western blots for STAT5 expression and other proteins was done as described in Materials and Methods. Right, western blots showing that pAKT is downregulated by momelotinib, whereas ERBB3 expression is not activated by the treatment with both trametinib or momelotinib. **b** Western blots showing that momelotinib or ruxolitinib treatment leads to the activation of ERKs signaling in different CRC cell lines. **c** Left, western blots showing that trametinib treatment down modulates CD15/FUT4 through inhibition of ERK activation. Right, western blot showing that STAT3 is activated by trametinib treatment in SW480 CRC cells. **d** The dataset GDS5029 confirms that other MEKi (selumetinib) did not affect the LY6G6D/STAT5 axis, *$P<0.05$ **$P<0.01$ by student *t* test.
